# Supplementary material for: The interactome of intact mitochondria by cross-linking mass spectrometry provides evidence for coexisting respiratory supercomplexes
Source: Mol Cell Proteomics. 2017 Dec 8;17(2):216–32. doi: 10.1074/mcp.RA117.000470 (PMC5795388; doi:10.1074/mcp.RA117.000470)
Supplement: Supplemental Data [file supp_RA117.000470_133922_0_supp_23978_fzffwf.zip › spectra_annotation/mito_spectra_annotation/318-1-180-1-15-1.pdf]

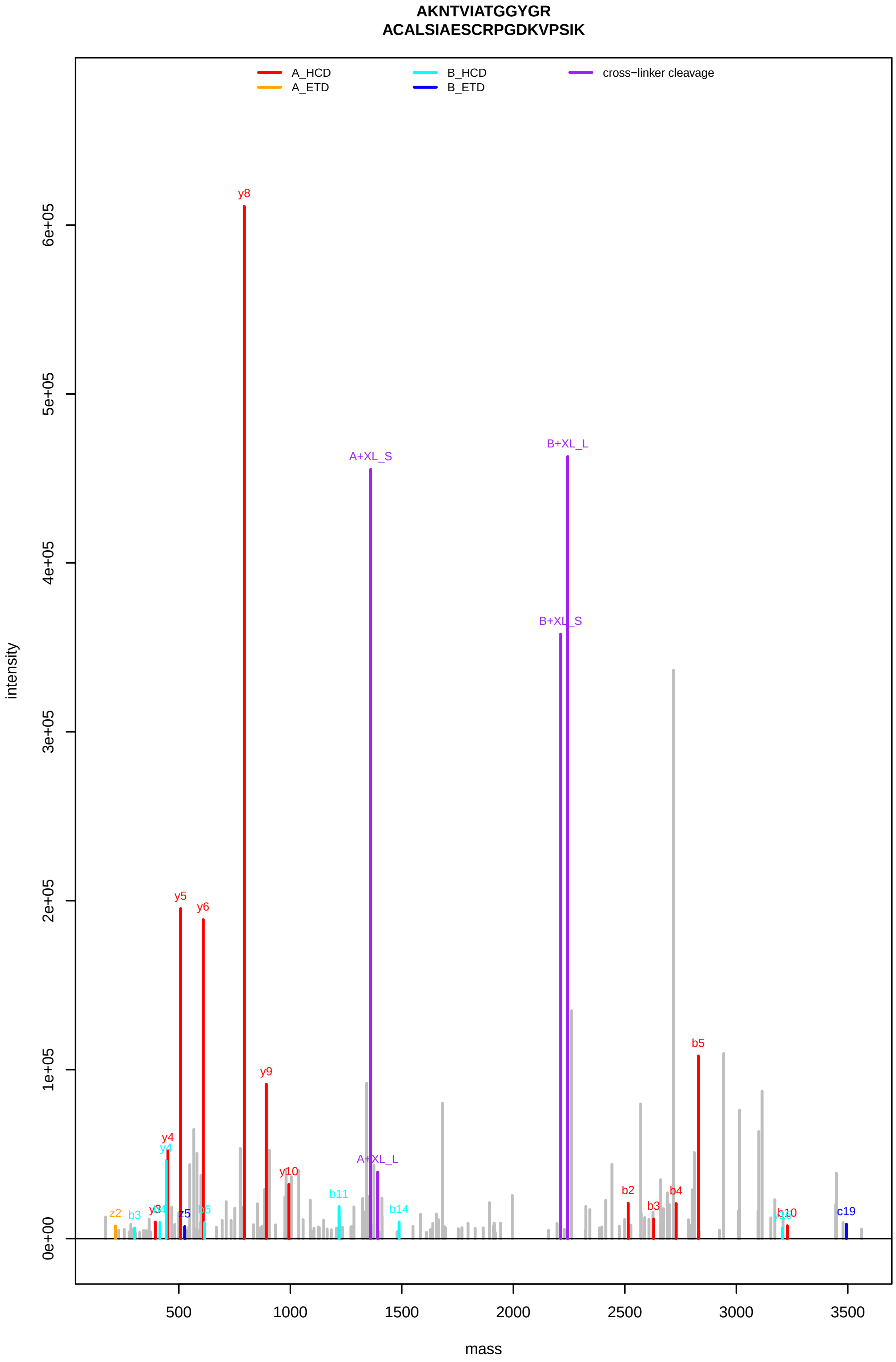

AKNTVIATGGYGR+XL_S

AKNTVIATGGYGR+XL_L

ACALSIAESCRPGDKVPSIK+XL_S

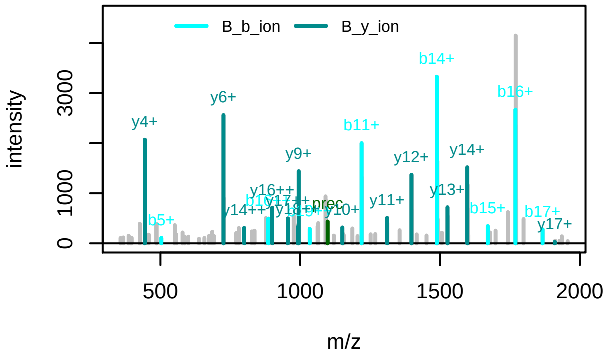

ACALSIAESCRPGDKVPSIK+XL_L
